# Precision measurement of rehabilitation interventions—a secondary analysis of motor error in a clinical trial with young children with cerebral palsy

**DOI:** 10.3389/fped.2024.1457329

**Published:** 2024-09-30

**Authors:** Julie C. Skorup, Samuel R. Pierce, Athylia C. Paremski, Morgan Alcott, Laura A. Prosser

**Affiliations:** ^1^ Department of Physical Therapy, The Children's Hospital of Philadelphia, Philadelphia, PA, United States; ^2^ Division of Rehabilitation Medicine, The Children's Hospital of Philadelphia, Philadelphia, PA, United States; ^3^ Department of Pediatrics, Perelman School of Medicine, University of Pennsylvania, Philadelphia, PA, United States

**Keywords:** cerebral palsy, motor function, motor error, precision rehabilitation, intervention

## Abstract

**Introduction:**

The delivery of precision medicine in rehabilitation will require not only precise measurement of participant response, but also precise measurement of the “ingredients” of intervention and their dose. As an example, we report the measurement of motor error in two treatment groups from a randomized controlled trial in toddlers (mean age 26.3 months) with cerebral palsy (CP). Our objective was to measure the type and amount of motor error during physical therapy sessions in young children with CP.

**Methods:**

Participants were stratified by motor function and age and randomly allocated to “conventional” physical therapy that generally prevented falls or to an intervention that encouraged error experience by not preventing falls (experimental group). Baseline motor and cognitive function were measured using the Gross Motor Function Measure-66 (GMFM-66) and Bayley 3 cognitive subscale (B3-C) prior to randomization. Randomly selected video recorded therapy sessions were manually coded to identify losses of balance defined as *falls* (child contacted floor), *rescues* (therapist prevented fall) or saves (child recovered their balance independently).

**Results:**

Average number of losses of balance per session were higher in the experimental group than the conventional group due to significantly greater falls. Saves were infrequent in both groups but were also significantly higher in the experimental group. Average number of rescues did not differ between groups. In the experimental group, greater frequency of falls was significantly related to GMFM-66. In both groups, greater frequency of saves was related to GMFM-66. Neither total losses of balance per session nor rescues were related to GMFM-66 in either group. There were no significant relationships between losses of balance and baseline cognition in either group, except greater frequency of saves was related to higher cognitive ability in the experimental group.

**Discussion:**

Our observations suggest that motor error experience is lower in toddlers with CP compared to peers with typical development but can be manipulated to higher doses of error during therapy sessions. Future work should investigate the relationship between type and amount of error experience and rehabilitation outcomes, as well as other “ingredients” of rehabilitation therapy. Tools to automate the precise measurement of intervention content are necessary for broad scale implementation.

## Introduction

Precision medicine is advancing health care from traditional “one-size-fits-all” models to care plans that consider patients’ individual needs including genetic, biomarker and/or psychosocial characteristics (1). In the pharmaceutical field, the *active ingredients and dose* of medicines are precisely adjusted to optimize the therapeutic effect. In pediatric rehabilitation, some “one-size-fits-all” models exist, but care is more commonly delivered on a trial-and-error basis, considering age, medical condition, level of impairment/disability, and social/contextual factors (2, 3). Similar to pharmaceutical prescription, precision rehabilitation will require the systematic study of how to adjust the *active ingredients and dose* of behavioral therapies to optimize the therapeutic effect.

Our ability to generate evidence from the iterative trial and error process (evaluate–treat–re-evaluate–adjust) has been complicated by the immense heterogeneity of clinical presentation in children and inconsistency in the selection of outcome measures and treatment regimens across patients. There are often multiple active ingredients in therapy as well as a variety of personal and environmental factors that influence treatment response. It is often difficult to identify the most important ingredients and how they interact with one another to optimize outcomes. Not surprisingly, our patients demonstrate wide variability in treatment response (3–6). Precision rehabilitation efforts to date have largely focused on the measurement of participants and their response to intervention, including standardizing measures of function and quantifying variability between participants to facilitate subgrouping of patients who share similar characteristics (7). These efforts are critical to the understanding of participant response, but equally critical to the delivery of precision rehabilitation is the need for detailed measurement of interventions (8, 9).

Here, we present an example of the precise measurement of an ingredient in pediatric rehabilitation therapy. We measured the frequency and type of motor error, specifically the inability to maintain postural control which leads to a loss of balance, experienced by participants in a randomized clinical trial (NCT02340026) in toddlers with cerebral palsy (CP) (10). CP is the most common childhood motor disorder. Children with CP have impairments in motor control due to a brain injury at birth or early in life, resulting in impairments in postural control, motor skill and functional limitations, often accompanied by other comorbidities such as impaired cognition and communication (11). Children with CP often begin early intervention therapy at a young age, some within the first months of life. While studies measuring the effect of different intervention approaches are becoming more numerous (3), there is a great deal of variability in the delivery of most interventions and detailed investigations on how to deliver precise protocols to optimize motor outcomes for individual children are lacking. A recent systematic review reported that therapy to improve motor control should encourage child-initiated movement, targeted motor training and incorporate task-specific and context specific activities at a high repetition and intensity (12). While useful as guidelines for therapists, these recommendations are general, are not precise prescriptions, and do not offer clear predictions for resulting neuroplastic change and motor outcomes.

Motor error, which is defined as the difference between the goal of the behavior and the actual motor outcome (13), may be an important ingredient when learning new motor skills and for acquisition of complex mobility skills. The nervous system constantly uses movement error information to adapt current movements and modify movement strategies for future movements. Motor error is highly prevalent in typically developing infants when learning to move (14) and has been studied in several rehabilitation applications in adults (15, 16). Typically developing novice walkers aged 12- to 19-months-old fall 31.5 times per hour, which decreases over the first months of walking experience to half this amount (14), and falling has been hypothesized as an important ingredient in learning to walk (17). In adults with hemiplegia from stroke, Hornby and colleagues reported diminished long-term gains in gait kinematics when postural and lower extremity errors were eliminated from practice (18). In pediatric rehabilitation, infants at risk for CP experienced a significantly lower amount of error during robotic crawl training compared to their peers with typical development (TD) (19).

The most common type of error during upright motor practice is a loss of balance. Without assistance, a loss of balance results in a fall or a self-recovery of balance to prevent falling. Young children with CP may not experience motor error as frequently as their peers because of their lower physical activity level or a limited ability to produce movements that challenge their balance. However, error experience has not been quantified in this population during free play or therapy sessions. Additionally, conventional therapy has typically focused on providing task specific practice of motor skills with hands-on guidance of motor patterns to prevent errors and the development of typical motor patterns. For example, many studies have focused on gait training interventions to promote independent walking and improve walking patterns in children with CP. These studies typically focus on providing guidance or external support during gait training, which limits error and prevents falls (20–23). Also, falls in children have traditionally been avoided to reduce the risk of injury (24) and some rehabilitation studies focus on strategies to prevent falls in children rather than encourage them (22, 25, 26). For these reasons, our objective was to measure the type and amount of motor error during physical therapy sessions in young children with CP as part of quantifying key ingredients of rehabilitation intervention.

## Methods

### Study design

This was a secondary behavioral video coding analysis of physical therapy sessions from the iMOVE (Intensive Mobility training with Variability and Error) clinical trial (NCTBLINDED) (10). The iMOVE study was a single-blind randomized controlled trial that compared the outcomes of a therapy program intended to provide motor learning experiences more similar to typically developing children that, among other variables, aimed for high rates of error experience (iMOVE group) to dose-matched conventional physical therapy that limited error experience (CONV group). The intervention phase was a minimum of 12 weeks, and participants’ caregivers could choose to extend the intervention to 18 or 24 weeks in duration. Therapy was planned for three 30-min sessions per week while actual attendance averaged 2.3 (SD 0.45) sessions per week. Two randomly selected therapy sessions per month, one from the first half and one from the second half of the month, were selected for secondary behavioral coding analysis.

### Setting

The study was conducted at a single site—the Children's Hospital of Philadelphia, which is a large urban pediatric academic medical center.

### Study sample

Participants were 12–36 months of age at enrollment, had the cognitive ability to follow one-step commands, had a diagnosis of CP or suspected CP [defined as motor percentile rank less than the 10th percentile on the Bayley Scales of Infant and Toddler Development, Third Edition (27, 28), and a neurological sign associated with CP, such as spasticity or periventricular leukomalacia] and had the ability to initiate pulling to stand at a surface as indicated by a score of 1 on the Gross Motor Function Measure (GMFM) item 52 (29). Participants were ineligible for the trial if they demonstrated any of the following: secondary orthopedic, neuromuscular or cardiovascular condition unrelated to CP, general muscle hypotonia without other neurological signs associated with CP (30), independent walking ability as indicated by a score of 3 on GMFM item 69, or history of surgery or injury to the lower extremities in the past 6 months. Sample size estimation for the clinical trial was based on pilot data that suggested evaluable group sizes of 17 participants in each would detect sufficient difference in gross motor function. We anticipated a uniform attrition rate of 20% to arrive at the target of 42 participants. After the baseline assessment, participants were stratified by motor ability and age and randomized to either the iMOVE or CONV treatment group (allocation ratio 1:1). The randomization list was prepared by the study statistician and stored in a secure electronic file accessible by the study coordinator.

### Interventions

All therapy was delivered by three experienced pediatric physical therapists who had 3–4 years full-time experience at the start of the study. The therapists were trained on characteristics of each therapy group, including strategies to minimize or encourage motor error during therapy, through a half-day workshop and supplemented by video review of pilot study sessions. Distinguishing characteristics of each group are detailed in the iMOVE protocol manuscript (10) and are summarized here. *iMOVE Therapy***.** The experimental therapy group was designed to mimic typical toddler motor learning experiences allowing for motor error experiences or losses of balance. Losses of balance were characterized as a fall (child contacts the ground), rescue (therapist interferes to prevent the child from contacting the ground) or save (child prevents self from contacting ground). Participants in this group received dynamic weight support (using the ZeroG® Gait and Balance training system, Aretech LLC, Ashburn, VA) during all therapy time to promote independent practice and the practice of challenging motor skills. The dynamic support system vertically unloads the user to provide a consistent level of unweighting despite how the user moves in vertical or horizontal space (31), allowing typical infant/toddler movements such as moving between the floor and standing, turning around and crawling, all while continuously unweighted. The system does not assist with maintaining balance. Infants may lose their balance and fall in the weight support system just as they might outside of it. The therapists provided supervision throughout the intervention to maintain a safe play environment and intervened during losses of balance to ensure the infants did not cause serious harm to themselves, such as hitting their head during a fall. *Conventional Therapy**.*** The CONV therapy group received traditional, therapist-directed pediatric physical therapy. Therapy focused on gross motor function and early gait training strategies with encouragement of “typical” movement patterns and manual guidance or correction to prevent error experience. Therapy activities were performed in blocks of practice, with the specific activities and level of therapist assistance tailored to each child.

### Functional assessment

An experienced physical therapist researcher, who was blinded to group assignment, collected baseline measures of gross motor function and cognition. Gross motor function was measured by GMFM-66 score, a Rasch-analyzed measure of gross motor function designed for children with CP (32). Cognition was measured by the percentile rank of the Bayley Scales of Infant and Toddler Development—Third Edition, cognitive subscale (27).

### Behavioral video coding

A primary coder watched each video continuously to identify all losses of balance that occurred during the therapy session using Datavyu software (33). The videos had been previously coded for activity (34). Losses of balance were identified within the specific activity that the child was performing (i.e., crawling, standing, walking) and were further coded as *falls*, *rescues*, or *saves.* A fall was defined as a loss of balance that results in the child hitting the floor or a surface without interference from the therapist to change the experience of the loss of balance. A rescue was coded when the child loses their balance but the therapist intervenes to prevent the child from falling to the floor. A save was coded when the child loses their balance but recovers it independently to prevent a fall.

### Reliability

Two individuals completed video coding for each therapy session. Coders were a physical therapist and a research assistant. Data reliability was ensured with a two-step process. First, and most important, all coders were trained on previously coded videos and achieved 80% agreement with prior coders before generating new data for analysis. Second, to maintain reliability among trained coders, each video was coded in full by a primary coder. Ten randomly selected loss of balance events were selected for secondary coding. If the primary coder identified <10 loss of balance events during a therapy session, other timepoints in the session (not identified by the primary coder as having a loss of balance event) were randomly selected to ensure that the secondary coder had a minimum of 10 events per session to code. We set an 80% agreement standard meaning that data from the primary coder was used for analysis for all videos that met or exceeded 80% agreement between coders. Agreement for events that do not have a loss of balance event was reached if the secondary coder counted 0 for losses of balance. For individual videos that did not reach 80% agreement, primary and secondary coders met to reconcile disagreements. The reconciled data from the double-coded portions were used for analysis, along with, if applicable, the primary coder's data on any remaining (single-coded only) portions of the video.

### Statistical analysis

Counts of each type of loss of balance were calculated and averaged across all coded sessions for each participant. Differences between groups in total losses of balance and each type of loss of balance were assessed with Mann Whitney tests with Bonferroni correction for multiple comparisons. Relationships of losses of balance to baseline levels of gross motor (GMFM-66) and cognitive function (B-3) were quantified by Pearson or nonparametric Spearman correlation as appropriate. Log 10 scale was used for visualization of cognitive scores due to skewing of data toward low end of range.

## Results

### Participant sample

The enrollment target of 42 participants was met. The 37 participants who completed the treatment phase of the trial were included in the analyses [mean age 22.1 months, Gross Motor Function Classification System I–IV (35)]. Four participants did not complete the treatment phase and one participant did not complete baseline assessment. See Table 1 for demographic characteristics of the sample. Fifty-six percent of participants were male, which reflects the population of individuals with CP (36). Ten percent of participants were Hispanic in ethnicity. Racial composition of the sample was 57% white, 24% black, 5% Asian, 11% mixed, and 3% not reported. The race and ethnicity of the sample reflects the diversity of the city of Philadelphia and surrounding region (37) and approximates the population of children with CP in the United States (38).

**Table 1:** Demographic and baseline characteristics of the participant sample. Age is reported as mean (SD) and was adjusted for preterm birth if applicable. Sex, ethnicity, race and gross motor function classification system level are reported as counts (% of total).

Age (months)	22.1 (6.5)
Sex	
Boy	20 (54.1%)
Girl	17 (45.9%)
Ethnicity	
Not Hispanic	32 (86.5%)
Hispanic	4 (10.8%)
Not reported	1 (2.7%)
Race	
White	21 (56.8%)
Black	9 (24.3%)
Asian	2 (5.4%)
Asian + White	2 (5.4%)
Black + White	1 (2.7%)
American Indian + Black + White	1 (2.7%)
Not reported	1 (2.7%)
Gross motor function classification system level	
I	5 (13.5%)
II	12 (32.4%)
III	11 (29.7%)
IV	9 (24.3%)

Participants were evenly assigned to the conventional (*n* = 19) and iMOVE (*n* = 18) treatment groups. As expected, due to stratification in randomized group allocation, there were no group differences in age (adjusted for preterm birth if applicable, *p* = 0.227), cognition (*p* = 0.637) or gross motor function (*p* = 0.969) at baseline. There were also no group differences at baseline in ethnicity (*p* = 0.581), race (*p* = 0.271) or sex (*p* = 0.756). A consort figure was published in the primary results paper for the iMOVE clinical trial (10).

A total of 422 videos were coded from the 37 participants. Thirty-three participants completed 24 weeks of therapy (each contributing 12 videos), one completed 18 weeks of therapy (contributing 9 videos) and three completed 12 weeks of therapy (two contributing 6 videos each and one contributing only 5 videos due to many missed sessions for medical complications).

### Reliability

Reliability for 385 videos (91.2%) exceeded the agreement standard of the primary and secondary coder of 80%. Consensus between the two coders on disagreements was reached for the remaining 37 videos (8.8%).

### Type and amount of error

With the iMOVE and CONV groups combined, there was an average of 12.2 (SD 6.6) losses of balance per therapy session, including falls, rescues and saves. The most frequent type of loss of balance were rescues (6.0, SD 3.2) followed by falls (5.3, SD 4.9) with saves occurring infrequently (0.9, SD 1.1). No serious injuries or need to stop a therapy session occurred from losses of balance. Minor bumps caused by loss of balance onto a nearby toy or bench were infrequent (3 total during all therapy sessions). Table 2 reports the total error and type of error for each group.

**Table 2:** Number and type of losses of balance per session for the iMOVE and conventional (CONV) therapy groups, reported as average counts (SD) per 30-min therapy session.

	iMOVE	CONV	Combined
Falls	8.6 (5.1)	2.2 (1.4)	5.3 (4.9)
Rescues	6.4 (3.8)	5.8 (2.7)	6.0 (3.2)
Saves	1.4 (1.3)	0.3 (0.5)	0.9 (1.1)
Total losses of balance	16.4 (6.4)	8.3 (4.0)	12.2 (6.6)

**Figure 1:**
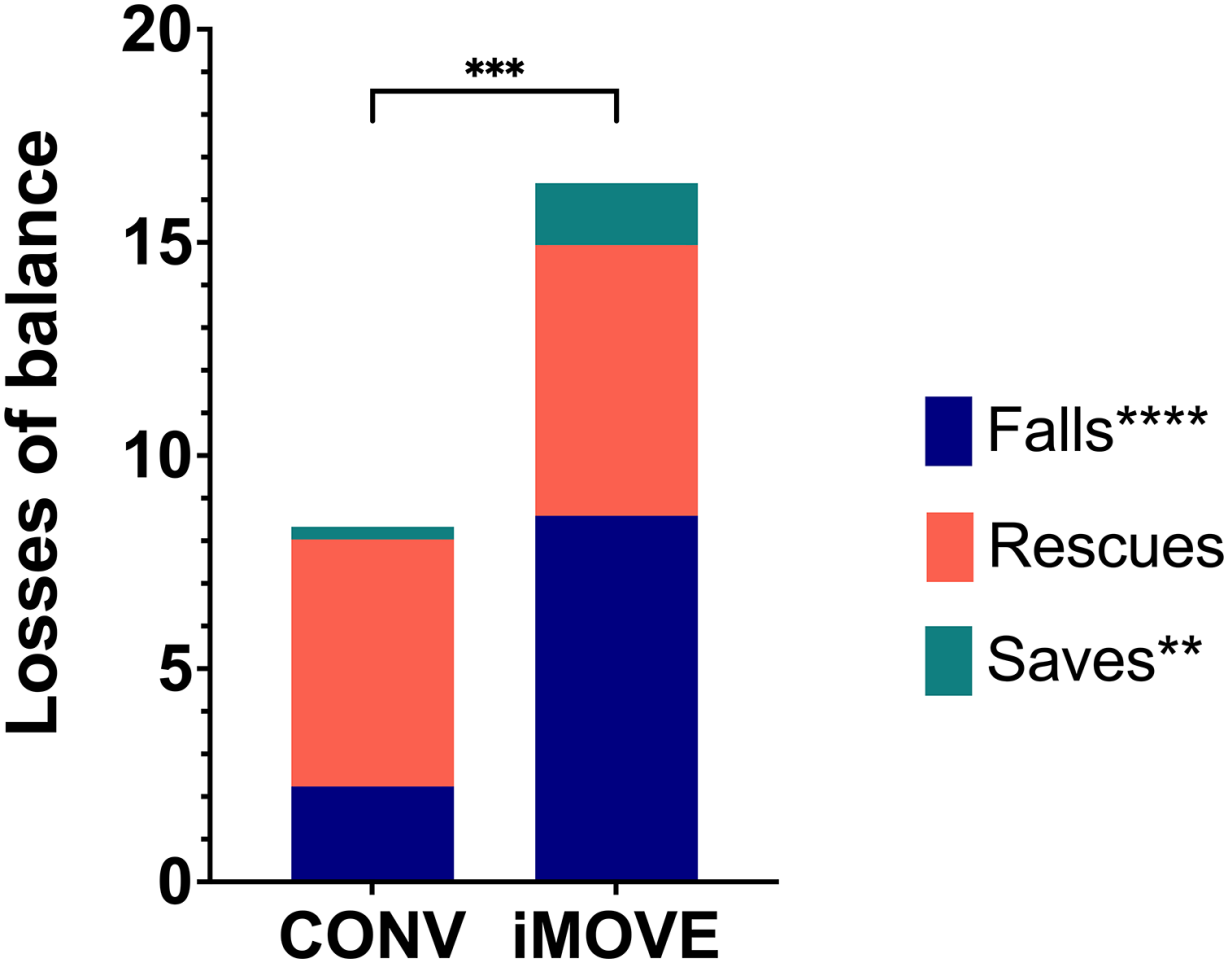
Stacked column graphs showing average losses of balance by type and group. Total losses of balance were higher in the iMOVE therapy group compared to the conventional therapy (CONV) group (*p* = 0.0003). More specifically, falls (*p* < 0.0001) and saves (*p* = 0.0064) were significantly different between groups. Rescues did not significantly differ between groups (Bonferroni corrected *p* = 1).

Total losses of balance (falls, rescues and saves) were higher in the iMOVE group than in the conventional therapy group, as intended. There was an average of 16.4 (SD 6.4) losses of balance per session in the iMOVE group compared to 8.3 (SD 4.0) in the conventional group. This difference is largely attributed to a significantly larger number of falls in the iMOVE group (*p* < 0.0001) with an average of 8.6 falls per session (SD 5.1) compared to 2.2 (SD 1.4) per session in the conventional group. Saves were infrequent in both groups, but they were significantly higher in the iMOVE group (1.4, SD 1.3) compared to conventional (0.3, SD 0.5; *p* = 0.0064). There was no difference in rescues between groups (Bonferroni corrected *p* = 1) with an average of 6.4 (SD 3.8) rescues per session in the iMOVE group and 5.8 (SD 2.7) rescues in the conventional group. Figure 1 displays the average losses of balance by type and group.

### Relationship with gross motor ability and cognition

**Figure 2:**
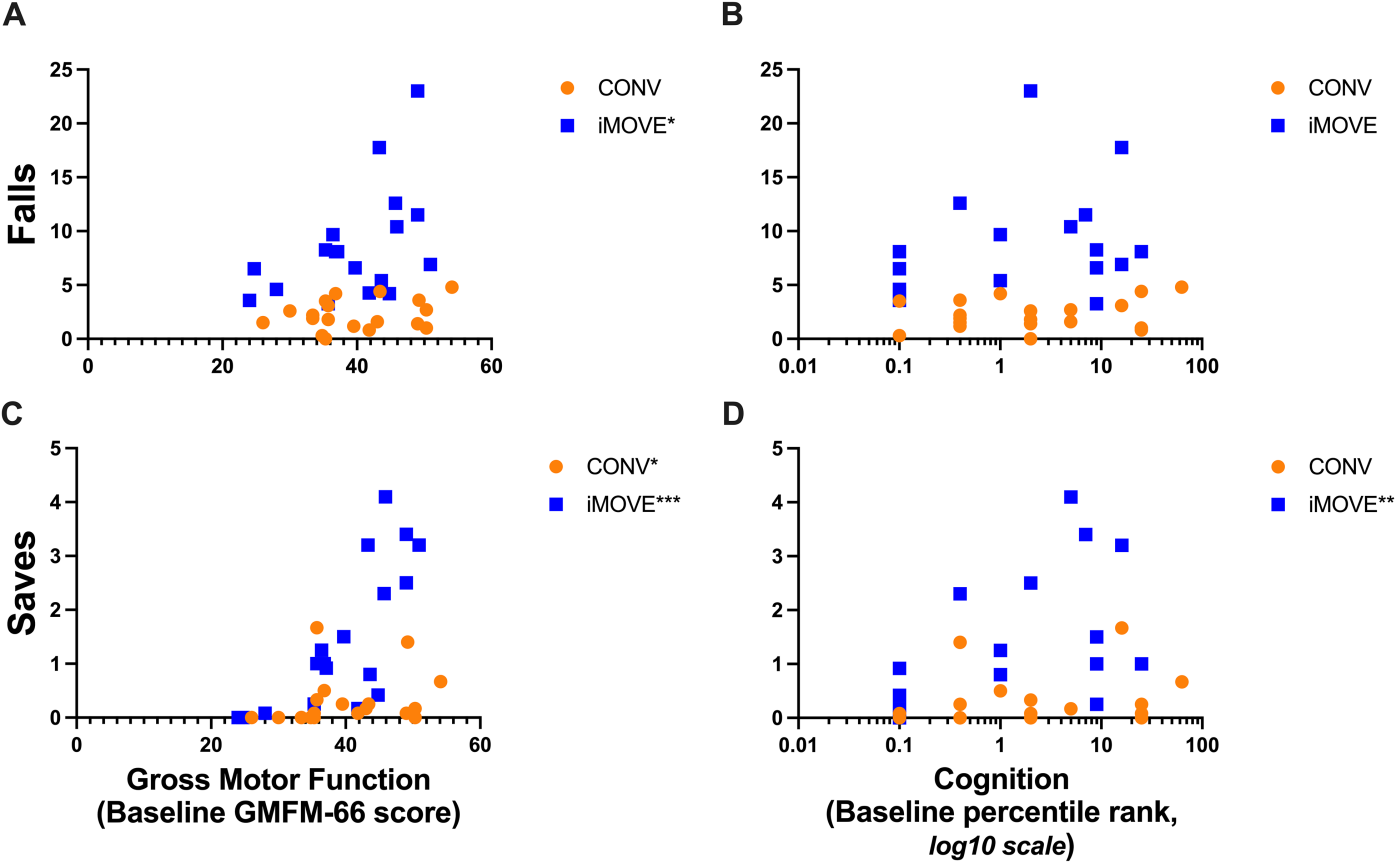
Scatter plots showing the relations between falls and saves during therapy sessions with gross motor function and cognition. Data points from participants in the conventional therapy group are orange. Data points from participants in the iMOVE therapy group are blue. **(A)** *More falls were related to higher gross motor function* in the iMOVE group (*r* = 0.51, *p* = 0.317*), but not the conventional therapy group. **(B)** *Frequency of falls was not significantly related to cognition* in either group. Log 10 scale used for visualization due to skewing of data toward low end of range. **(C)** *More saves were related to higher gross motor function* in both groups (iMOVE *r* = 0.78, *p* = 0.0001***; conventional *r* = 0.55, *p* = 0.014). **(D)** *More saves were related to higher cognition* in the iMOVE group (*r* = 0.63, *p* = 0.0049**), but not the conventional therapy group. Log 10 scale used for visualization due to skewing of data toward low end of range.

The average losses of balance per session was not related to baseline gross motor function for either the iMOVE or CONV groups (iMOVE *r* = 0.27, *p* = 0.284; CONV *r* = 0.34, *p* = 0.158). However, the number of falls was related to higher gross motor function in the iMOVE group (*r* = 0.51, *p* = 0.317) but not in the conventional group (*r* = 0.24, *p* = 0.326). Rescues were not related to baseline gross motor function in either the iMOVE or conventional therapy group. The frequency of saves was related to baseline motor function for both groups (iMOVE *r* = 0.78, *p* = 0.0001; CONV *r* = 0.55, *p* = 0.014), with those who were higher functioning at baseline on the GMFM-66 demonstrating higher ability to save their balance than those who were lower functioning at baseline. Total losses of balance were not related to baseline cognition for either group (iMOVE *r* = 0.11, *p* = 0.652; CONV *r* = 0.22, *p* = 0.360). The frequency of falls was not related to baseline cognitive function in either group (iMOVE *r* = 0.39, *p* = 0.109; CONV *r* = 0.14, *p* = 0.560). The frequency of saves was related to baseline cognitive function in the iMOVE group (*r* = 0.63, *p* = 0.014) with the participants with higher baseline cognitive ability demonstrating more saves during therapy, but not in the conventional group (*r* = 0.31, *p* = 0.194). Figure 2 displays the average number of falls and saves per session for each child relative to their baseline gross motor function and cognition. Rescues are not shown because they did not differ between groups and were not related to baseline gross motor or cognitive function.

## Discussion

This is the first study to report the frequency and type of motor error during rehabilitation therapy sessions. As intended by the study design, the iMOVE therapy group experienced more overall motor error than the conventional therapy group due to a greater number of falls. While saves were also greater in the iMOVE group, the number of rescues were not different between groups. This suggests that toddlers with CP do experience losses of balance during therapy sessions when they are practicing new skills and supports the hypothesis that toddlers with CP do not traditionally experience falls similar to the levels of their typically developing peers, perhaps because they are unable to generate motor experiences that challenge their motor skill or balance. Importantly, we showed that losses of balance can be manipulated to encourage greater fall experience during therapy.

Not surprisingly, children with CP experience less falls than their typically developing peers. Typically developing toddlers between the ages of 12- and 19-months fall an average of 17 times per hour during free play and early walkers >31 times per hour (14). With a focus on error-based practice, toddlers with CP are able to experience falls at similar rates as their typically developing peers (16.4 times per 30-min session, if maintained would be 32.8 times per hour) (14). However, it is not yet known if an increase in error experience alone will result in better motor outcomes in children with CP.

While error experience appears to be an important component of motor learning and neuromotor rehabilitation, several different strategies to manipulate error during rehabilitation have been proposed for different neurological populations. Error augmentation is an example of one technique that magnifies the degree of error experienced by the patient with the goal of forcing the patient to “fight” the error signal to correct the movement. The exaggerated feedback may make the error more noticeable to the patient, especially small errors amplified as larger ones, which is hypothesized to improve learning (39–41). The relative size of errors for functional gain needs further study because excessively large errors have been reported to prohibit learning (42). For instance, in a study where participants walked on a split belt treadmill and encountered errors, the larger errors that were created due to environmental obstructions prevented the transfer of motor learning off the treadmill to overground walking (15). Additionally, a systematic review of error augmentation during upper limb motor training in adults after stroke showed some evidence that error augmentation is beneficial, but the findings are not conclusive due to imprecise measurements and variable dosing. This underscores the need for more precise ways to measure rehabilitation interventions to quantify the dose of active ingredients being prescribed (39).

In contrast to error augmentation, error reduction strategies, such as errorless learning, have been explored in adults with cognitive impairment (43). In errorless learning paradigms, error is prevented during the practice of a task and may involve breaking the task down to easier steps, modeling the task before allowing practice, and providing immediate correction to decrease motor errors (44). In adults with traumatic brain injury, errorless learning showed better learning in discrete task specific activities (45), but questionable generalization to more complex tasks in changing environments (46). In children with cognitive impairment, reduction of error during learning decreases the cognitive demands associated with learning a new task which may result in improved motor learning, especially for more complex tasks (47, 48). Maxwell and colleagues showed that reducing error during a complex task in children was more beneficial in children with lower motor abilities (49). This could suggest that minimizing error in children with cognitive impairments, similar to some of the participants in our study, may result in improved learning of motor skills for some children. These hypotheses need further study in specific subgroups of children based on age and cognitive function.

The approach we used encouraged a higher frequency of motor error but did not manipulate (augment or minimize) the magnitude of error. Instead, the error experience was caused by the child's own postural instability or movements. This type of approach is most consistent with an “error-based” strategy like the one used by Torres-Oviedo using a split belt treadmill paradigm to measure the influence of error during a walking task on the ability to translate to walking overground (15). They found that natural error produced by one's own body (i.e., error produced by one's own postural instability or movement pattern error) resulted in better motor learning and transfer to adapted walking off the treadmill compared to error that was abrupt and caused by the environment (i.e., large and sudden change in speed of split belt treadmill) (15). And while it is suggested that error during motor skill acquisition is an important component of acquiring the ability to walk (17), the specific type and amount of error that can produce learning and generalizability to more advanced motor skills in children with CP needs further study.

Our results show that type and dose of error can be manipulated during physical therapy sessions in toddlers with CP who have cognitive and motor impairments. But it is important to consider if a child with brain injury is able to adapt motor performance from error experience. Studies have shown that adults who experienced a stroke were able to adapt from error but required more practice before error-free movement occurred (16). When comparing higher error vs. error minimizing strategies in throwing tasks in children with CP, children learned with both approaches with no difference in motor learning between the approaches (50). Additionally, neither error augmentation nor error minimization was superior in improving spatiotemporal gait parameters in adults post stroke when walking on a spilt belt treadmill (51), which raises the question of whether type of error is more important in some applications but not others. Levac and colleagues showed that a gradual increase in error may be best for retention of skills, starting with errorless learning to allow the participants to strengthen their motor skill before having to adapt movement in response to error. This hybrid approach to error that is modified based on the individual's motor and cognitive function is promising and should receive further study (52).

There are many factors to consider how children with CP make gains in their motor skills and it is important to study how these factors interact with one another to improve functional outcomes. For instance, in our study, we found that baseline cognition was related to the number of saves the child was able to produce in the iMOVE group only. It is unknown how cognition may affect the child's ability to learn motor skills, as well as learn from error experience during rehabilitation. Children with higher baseline cognition may perform higher motor skills and be able to adapt their movement in response to error, such as produce a save from a loss of balance. The thresholds for cognitive and motor skills that allow children with CP to practice and learn from error for benefit is unknown. It is also important to consider that the ability to capture true cognitive capabilities in this population is limited because the assessment of cognition requires fine motor control or speech, and often vision and hearing capacity, all of which can be impaired in this population. Additionally, there are other factors besides cognition, such as motivation to move, attention and parent involvement, that may contribute to rehabilitation outcomes but are difficult to precisely measure as well as manipulate.

Our investigation provides an approach that could be a model for application to other populations and other rehabilitation interventions. However, our approach has some limitations. First, error was manipulated and measured during therapy only and not at home or in children's natural environments. Future work should explore ways to manipulate motor error during play outside of therapy to allow more opportunities for adaption and learning of motor skills. In addition, visual video coding to identify error also requires the loss of balance to be large enough to be seen by the coder. This may result in missing the smaller occurrences of error, specifically error that the child is able to self-correct resulting in an undercount of saves. Finally, the amount of time to manually video code therapy sessions, including double coding the videos to ensure reliability, is considerable. The videos of the 30-min therapy sessions were first coded for gross motor activity (34) and then coded for losses of balance. This took hundreds of hours from multiple coders to complete, including the time and effort to train coders to reliability. Automating data capture will be essential to improving efficiency and consistency in quantifying the ingredients of therapy, such as motor error, and is needed for broad scale implementation.

Future research is needed to investigate the relationship between error and rehabilitation response as well as other ingredients of therapy and treatment response—which ingredients are essential to improve motor control and skill acquisition, including the minimum dose and the interaction between the ingredients for optimal change as well as how baseline characteristics and family resources can affect outcomes. Generating evidence-based dose-response trajectories for active therapeutic ingredients will inform the identification of thresholds for therapeutic effect in specific subgroups of patients and allow the prediction of individuals’ likelihood of response to specific treatment regimens.

Precision medicine in rehabilitation is an ambitious but important goal in neurorehabilitation, especially in pediatric therapy. Orthopedic rehabilitation has more readily been able to utilize protocols and clinical practice guidelines that offer precise details of examination, intervention and expected outcomes (53–56). In pediatric rehabilitation, there is a published clinical practice guideline for the physical therapy treatment of congenital muscular torticollis. It provides evidence for specific ingredients of therapy that must be included for a therapeutic effect, such as neck and trunk range of motion and environmental adaptations, as well as specific ways to measure outcomes to assess if therapy is indeed effective (57). While precision rehabilitation is increasing for orthopedic and muscular conditions, application to motor training interventions in infants and toddlers with CP is more difficult.

The ability to precisely time, dose and deliver interventions to improve motor control in young children with CP is important so that providers and families spend their limited time to maximize outcomes, with the ultimate goal being improvement in motor control and function for lifelong improvement in functional independence and motor outcomes. Equally important is identifying ingredients or doses that do not contribute to functional gains so families can focus on other priorities. Future work should focus on quantifying the many variables of intervention, and their automation, to identify precisely how to optimize rehabilitation outcomes with the minimum dosage.

## Data Availability

The raw data supporting the conclusions of this article will be made available by the authors, with execution of sufficient privacy agreements.
